# Familial Clustering of Adolescent Emotional Problems in a Nationally Representative Sample in Britain: A Multilevel Investigation

**DOI:** 10.1192/bjo.2024.178

**Published:** 2024-08-01

**Authors:** Kate Jordan, Kishan Patel, Ramya Srinivasan, Glyn Lewis

**Affiliations:** 1 UCL Division of Psychiatry, London, United Kingdom; 2 UCL MRC Unit for Lifelong Health and Ageing, London, United Kingdom

## Abstract

**Aims:**

The prevalence of emotional problems, such as depressive and anxiety disorders, increases sharply during adolescence. There is evidence for familial clustering of mental health problems during early childhood and adulthood, however no studies have investigated whether adolescent mental health problems cluster within families. This study tests the hypotheses that i) emotional problems during adolescence cluster within families, and that ii) conduct, peer and hyperactivity problems, prosocial behaviour and overall emotional and behavioural difficulties during adolescence also cluster within families.

**Methods:**

We used cross-sectional data from a nationally representative survey of UK households, collected between 2019 and 2021, with 4,088 participants aged 10–16 years. Analyses included 1,241 participants who had complete outcome data and complete data on all covariates of interest. The Strengths and Difficulties Questionnaire (SDQ) was used to examine emotional problems, as well as conduct, peer and hyperactivity problems, prosocial behaviour and total difficulties. Multilevel modelling was used to: estimate clustering of i) emotional problems and ii) conduct, peer and hyperactivity problems, prosocial behaviour and total difficulties, within families, after adjusting for several individual- and family-level covariates associated with adolescent mental health problems (including individual and family demographics, school and sibling bullying, quality of parent-child relationship, parent mental health and parent romantic relationship satisfaction).

**Results:**

After adjusting for known covariates of adolescent mental health problems, there was substantial clustering of adolescent emotional problems (ICC: 0.439; CI^95%^: 0.36–0.52; SE: 0.042) and overall adolescent emotional and behavioural difficulties (ICC: 0.417; CI^95%^: 0.34–0.50; SE: 0.043) within families. There was also evidence of clustering of adolescent peer problems (ICC: 0.374; CI^95%^: 0.28–0.48; SE: 0.051), hyperactivity (ICC: 0.332; CI^95%^: 0.25–0.42; SE: 0.044), prosocial behaviour (ICC: 0.263; CI^95%^: 0.18–0.37; SE: 0.048) and conduct problems (ICC: 0.232; CI^95%^: 0.14–0.35; SE: 0.053) within families after adjustment.

**Conclusion:**

We found strong evidence that adolescent emotional problems cluster within families even after accounting for individual- and family-level covariates which are associated with adolescent mental health problems. Over 40% of the variation was accounted for at the family level. This indicates how the contextual characteristics of the family environment may influence the mental health of young people. As such, social policy aiming to prevent or improve the mental health of young people should focus on family context.

